# Analyzing the Perspectives of Health Professionals and Legal Cannabis Users on the Treatment of Chronic Pain With Cannabidiol: Protocol for a Scoping Review

**DOI:** 10.2196/37697

**Published:** 2023-01-20

**Authors:** Priyanka Kumar, Charles Mpofu, Dianne Wepa

**Affiliations:** 1 School of Public Health Auckland University of Technology Auckland New Zealand; 2 University of Bradford Bradford United Kingdom; 3 University of South Australia Adelaide Australia

**Keywords:** CBD, cannabidiol, medicinal cannabis, medical cannabis, chronic pain, pain, perspectives, opinions, health professionals, medical professionals, users, medicinal cannabis users, medical cannabis users, New Zealand, NZ

## Abstract

**Background:**

Medical cannabis is one of the most commonly reported treatments for chronic pain. The wide acceptance and research in alternative medicine have put medical cannabis in the limelight, where researchers are widely examining its therapeutic benefits, including treatment of chronic pain.

**Objective:**

The purpose of this scoping review is to provide an overview of the perspectives on cannabidiol as an alternative treatment for chronic pain among health professionals and legal cannabis users.

**Methods:**

The framework of Arksey and O'Malley guides the design of this scoping review, and the elements reported use the recommended guidelines of the PRISMA-ScR (Preferred Reporting Items for Systematic Reviews and Meta-Analyses Extension for Scoping Reviews). A comprehensive literature search accessed the databases CINAHL Complete and MEDLINE via EBSCO, Australia/New Zealand Reference Centre, PsycINFO, Ovid Emcare, Wiley Online Library, Scopus, Informit New Zealand Collection, and Google Scholar for published literature, and then it was extended to include gray literature. Gray literature searches included searching the databases Australia/New Zealand Reference Centre, Informit New Zealand Collection, INNZ: Index New Zealand, ProQuest Dissertations & Theses Global, and AUT Tuwhera Research Repository, and the website nzresearch.org.nz. The studies included in this scoping review were assessed for eligibility for inclusion using the following criteria: published in English after 2000, conducted in New Zealand (NZ) or Australia, and aimed to investigate the perspectives of health professionals and medical cannabis users using interviews for data collection. Studies were screened for inclusion using Covidence, a software tool to filter search results, and the risk of bias was assessed using the Critical Appraisal Skills Programme tool. Although this is not a required step for scoping reviews, it added an element of strength to this scoping review. Data will be analyzed using thematic analysis guided by Braun and Clarke. The findings from the data analysis will be presented in a table, which will then inform the key themes for discussion.

**Results:**

The database search started in October 2021 and was completed in December 2021. The total number of studies included in this review is 5 (n=5). Studies included were conducted in NZ or Australia and examined the perspectives using participant interviews. This scoping review is anticipated to be submitted for publication in December 2022.

**Conclusions:**

Using perspectives is a valuable tool to understand the challenges experienced by health professionals and medical cannabis users associated with medical cannabis treatment. Addressing these challenges through interventions that are highlighted through perspectives such as educating health professionals to increase access to medical cannabis in NZ may aid in policy reformulation for medical cannabis in the context of NZ. Thus, this scoping review highlights the importance of medical cannabis research and suggests recommendations to guide and inform medical cannabis policy in the context of NZ.

**International Registered Report Identifier (IRRID):**

DERR1-10.2196/37697

## Introduction

### Overview

Medical cannabis is one of the most reported treatments for chronic pain [1,2]. Chronic pain is one of the most disease-burdening conditions in the world [3,4], which is estimated to affect 1 in 5 adults of the world’s general population [5]. Opioids are currently the preferred method of treatment, trusted by physicians to manage chronic pain [6]. However, although opioids are readily attainable and a preferred method of treatment, there is excessive use of opioids globally as more people access opioid prescriptions for chronic pain management [7]. The commonly reported reasons for researching opioid alternative treatments are opioid-related drug abuse, overdosing, associated risks, and high prescription rates [8].

Medical cannabis, or *Cannabis sativa* L. (*C sativa*), is a plant of the Cannabaceae family, which has properties that are particularly interesting to researchers for their therapeutic benefits as alternative treatments for varying chronic conditions including chronic pain [9]. It comprises 2 elements: delta-9-tetrahydrocannabinol (THC) and cannabidiol (CBD). THC is the psychoactive compound, consuming which produces a “high” as it alters the functioning of the central nervous system (CNS). CBD, however, tends to be the focus of modern-day research for its medical benefits when consumed in small doses. The exact mechanism of how CBD works in the body is unclear. However, it is known to activate the body’s internal endocannabinoids via the endocannabinoid system [10]. It is suggested that the neurotransmitters emitted from the CBD products bind to the cannabinoid receptors in the body’s CNS and activate signals. The activated signals cause signal blocking, which results in relief from discomfort caused by medical conditions [10].

### Background

In 2018, New Zealand (NZ) legalized medical cannabis by amending the Misuse of Drug Act 1975 to control the use of cannabis as medicine. In April 2020, the Medical Cannabis Scheme was implemented in NZ. The Medical Cannabis Scheme aims to allow qualifying users to access quality medical cannabis products. Currently, qualifying users are limited to only those diagnosed with multiple sclerosis [11]. However, chronic pain management is one of the more commonly proposed reasons to use medical cannabis [3,4]. While some studies show improvements when using medical cannabis for chronic pain [12,13], others have emphasized their apprehensions with study designs and research affiliations with pharmaceutical companies suggesting more positive applications [8,14-16]. Others explain that studies investigating the relationship between medical cannabis and chronic pain have a high prevalence of reporting bias, over-reporting of results, small sample sizes, short-duration outcomes, and short follow-up periods [8]. These concerns are further justified by similar concerns voiced by researchers who strongly argue for the need to expand research on medical cannabis and its use for chronic pain [16]. Overall, the findings from studies suggesting the effectiveness and efficiency of using medical cannabis as a treatment for chronic pain are considerably low, and therefore, further research is required [4].

### Rationale

In addition to literature highlighting the inconsistencies of medical cannabis as an alternative treatment for chronic pain, seeking the opinions of health professionals and medical cannabis users may add another dimension to this body of evidence. The studies exploring the perspectives of health professionals and legal cannabis users using a government-standardized CBD product are limited. Previously, studies explored the perspectives of health professionals and medical cannabis users using surveys without performing participant interviews. Therefore, this scoping review adds a methodological dimension too [17-19]. Furthermore, when these studies were conducted, medical cannabis was not legal in NZ. Therefore, these studies have not examined a form of standardized CBD treatment legal for use in NZ. This highlights a possible limitation as the participants would have reported outcomes consuming substances from the black market.

For this reason, study results cannot be directly linked to chronic pain treatment with CBD, considering the unknown concentrations of CBD, THC, and other contaminants in black market substances. The perspectives of individuals with chronic pain who use medical cannabis and the perspectives of health professionals who prescribe medical cannabis are essential and should contribute to the clinical, social, economic, and political discussion of medical cannabis in NZ. This scoping review aims to examine the perspectives surrounding medical cannabis use to treat chronic pain among health professionals and legal cannabis users. This scoping review is being performed as part of a qualification intended for achievement by the primary author (PK), which is not reported in this scoping review protocol.

### Overall Aim

This scoping review aims to provide an overview of the perspectives on CBD as an alternative treatment for chronic pain among health professionals and legal cannabis users.

The specific objectives are as follows:

1. To search and synthesize literature on the perspectives of health professionals and legal cannabis users in treating chronic pain with CBD
2. To provide literature that will add to the current knowledge that may inform policy regarding prescribing CBD treatment for chronic pain in NZ.

## Methods

### Overview

The framework of Arksey and O'Malley [20] guides the design of this scoping review, and the elements reported will use the recommended guidelines of the PRISMA-ScR (Preferred Reporting Items for Systematic Reviews and Meta-Analyses Extension for Scoping Reviews) [21]. Scoping reviews have become a popular tool for synthesizing evidence. Similar to a systematic review, a scoping review follows a structured process. A scoping study is practical when providing an overview of what is already known about a particular topic or in circumstances where the literature volume is broad and detailed. The most common reason to perform a scoping review is to identify and map the currently available evidence [20,22]. A useful aspect of performing a scoping review is when studies aim to identify themes and concepts reported and discussed in the literature on a particular topic. To address the purpose of this review, a scoping review is a better suited approach compared with a systematic review as it seeks to examine the perspectives of health professionals and legal cannabis users while identifying and mapping the currently available literature on medical cannabis and chronic pain [23].

### Developing the Research Question

The search protocol was developed using the PICO(T) tool, developed by Richardson and Wilson [24], which comprises the components People, Intervention, Comparison/Control, Outcome, and Time. Scoping reviews have broad questions, as the general aim of a scoping review is to map the literature available on a topic. Therefore, 2 elements of the PICO(T) tool were used to formulate a broad question sufficient to perform a scoping review. The 2 elements of the tool that were used in formulating the question for this scoping review are population/problem (P) and intervention (I) [25].

### Research Question

What are the perspectives of health professionals and legal cannabis users (population) about the use of medical cannabis (intervention) in treating chronic pain (problem)?

### Searching the Literature

The literature search was performed from October to December 2021. In the initial stages of searching, there was no restriction on searching by geography; however, because of the number of results that were present for each search, the supervisors were consulted to narrow the search to studies limited to NZ and Australia. To our knowledge, this is the first scoping review to explore the perspectives of health professionals and legal cannabis users restricted to NZ and Australia. The decision to restrict the literature search by geographical location was taken given the nature of the study to explore the perspectives of health professionals and legal cannabis users where studies were limited. This scoping review also aims to add knowledge through a wider qualification to aid policy changes in NZ. Therefore, it is vital to explore the perspectives of health professionals and legal cannabis users to ensure informed policy change decisions in NZ. As a result, the literature search for this scoping review was restricted to NZ and Australia. In addition, reviewing the availability of literature without a geographical restriction is beyond the capability of the primary researcher in the time allocated for the completion of the intended qualification.

The databases that were searched included the following: CINAHL Complete and MEDLINE via EBSCO, Australia/New Zealand Reference Centre (EBSCO), PsycINFO, Ovid Emcare, Wiley Online Library, Scopus, Informit New Zealand Collection, and Google Scholar.

In addition to the databases listed above, gray literature was searched using platforms dedicated to this purpose. The platforms that were used to search gray literature included the databases and website listed as follows: Australia/New Zealand Reference Centre, Informit New Zealand Collection, INNZ: Index New Zealand, ProQuest Dissertations & Theses Global, AUT Tuwhera Research Repository, and nzresearch.org.nz.

The keywords and phrases used to search were kept the same for consistency. The keywords were often combined with truncation and Boolean search techniques. An example of the search string is presented in Textbox 1, and the full search string can be found in Multimedia Appendix 1. All the keywords used to modify the search during the database, gray literature, and website searches are presented in Textbox 2.

**An example of the search string used to search databases, gray literature, and websites.**

**Example of the search string used to search**

CBD OR Cannabidiol* OR (Medical Cannabis) AND Pain OR (Chronic Pain) AND Perspective* OR Opinion* OR Experience* AND Professional* OR (Health professional*) AND (New Zealand) OR Aotearoa OR Australia

**Example 1 of the search string used to search websites**

Medical cannabis AND Pain

**Example 2 of the search string used to search websites**

CBD AND Pain AND New Zealand OR NZ

**The search terms that were used to perform the database and gray literature search**

**CBD**

CBD, Cannabidiol, Medical cannabis, Medicinal cannabis, Medical marijuana, Medicinal marijuana

**Pain**

Pain, Chronic pain, Neuropathic pain, Chronic condition, Non cancer chronic pain

**Perspectives**

Perspective, Opinion, Attitude, View, Experience, Lived experience, Knowledge User experience, Professional experience

**Population**

Professional, Health professional, Medical professional, User, Patient, Doctor, Nurse, Pharmacist

**Geography**

New Zealand, NZ, Aotearoa, Australia, Aus, Auz

### Eligibility Criteria

Eligible studies were those published in English between 2000 and 2021 and were either qualitative or mixed methods studies using interviews. The recruited participants were 18 years or older and used medical cannabis for chronic pain, or chronic pain in conjunction with another condition. Also, studies were eligible to be included if they were conducted in either NZ or Australia and must have aimed to investigate the lived experience, perspectives, or opinions of health professionals of all categories, for example, general practice, mental health professionals, nurses, and medical cannabis users, specifically CBD.

### Exclusion Criteria

Studies were excluded if they were conducted outside of NZ or Australia, did not use interviews, and used other methods of analyzing perspectives such as surveys. Studies focusing on medical cannabis for mental health–related disorders, those examining the efficacy of cannabis treatment, and recreational cannabis studies were also excluded from this review.

### Screening or Selecting the Studies

After the search was completed in each database, the search results were exported as an RIS file into the reference manager EndNote X9. After all search exports from each database were completed, a final XML file was exported from EndNote X9 and imported into Covidence, a web-based software tool useful when performing a type of comprehensive literature review [26]. The purpose of the software is to aid in screening studies, data extraction, and quality assessment required to perform a full review [26,27]. The software Covidence was used in all stages to screen titles and abstracts for the first screen, the full-text screening stage where studies were either included or excluded, data extraction, and quality assessment of the studies included. The primary researcher (PK) completed the initial screening and selected the articles to be screened in the abstract screening stage. The second reviewer, also the secondary supervisor in this research project (DW), also individually screened the titles for abstract screening in Covidence. Although an appraisal of the literature is not required to be included in a scoping review, articles included in our scoping review will be critically appraised using the Critical Appraisal Skills Programme (CASP) tool. Appraising literature that is included in the review will aid in further identification of research gaps.

The quality assessment was performed in Covidence. At this stage, the template provided by Covidence was customized to suit the data present in qualitative studies, adapting it to the CASP checklist for qualitative studies [28]. The CASP tool is a simple tool that can be used to appraise the strengths and limitations of qualitative research methodology. CASP is commonly chosen among novice researchers as the tool is user friendly and easy to understand. It was created for appraising health-related research and is endorsed by Cochrane and the World Health Organization for use in qualitative evidence synthesis [29,30]. It is for these reasons that CASP was selected as the most appropriate tool to be used in this scoping review.

**Figure 1:**
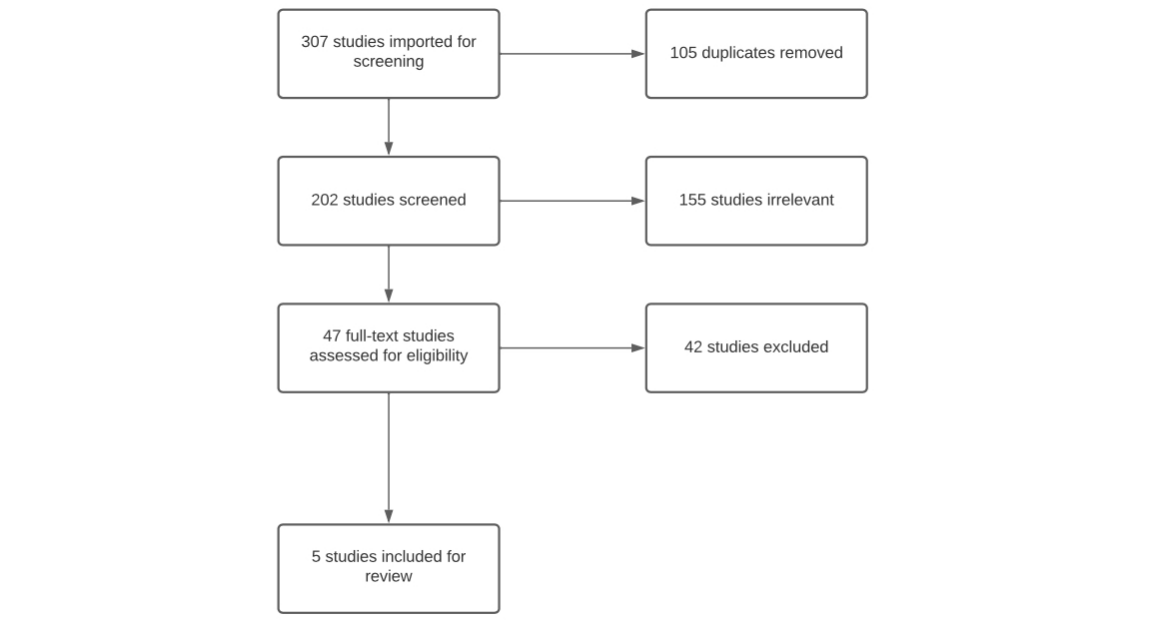
PRISMA-ScR (Preferred Reporting Items for Systematic Reviews and Meta-Analyses Extension for Scoping Reviews) flow diagram of the study selection process from databases.

**Figure 2:**
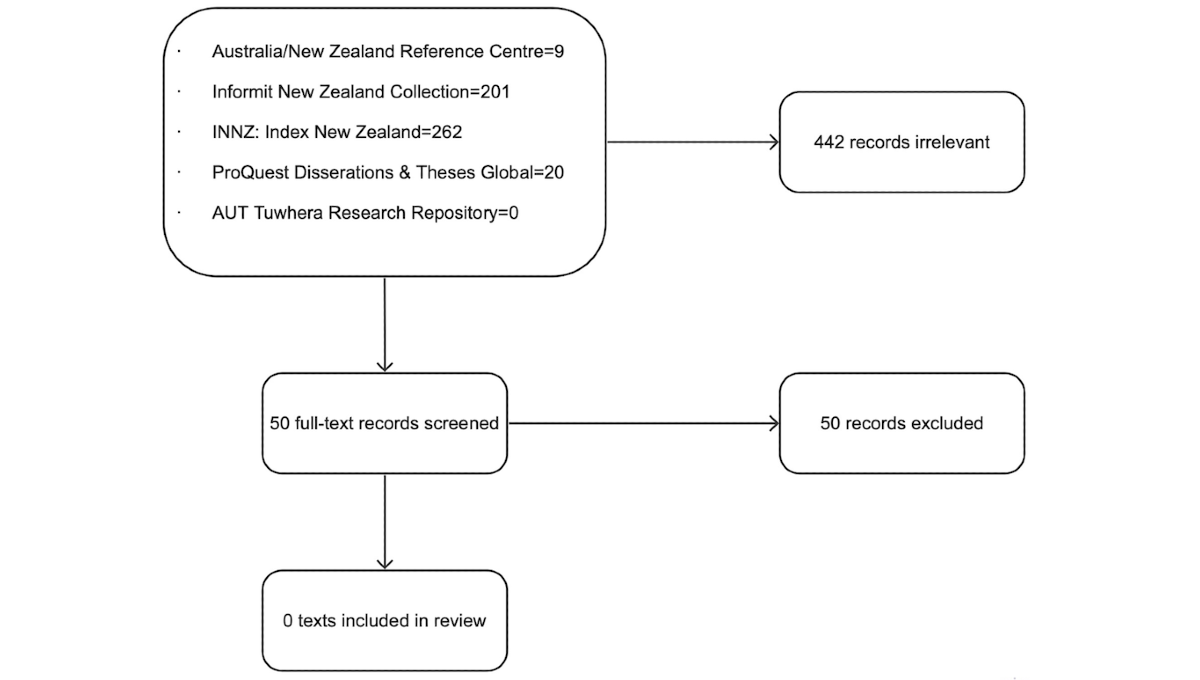
Flow diagram of the study selection process from gray literature.

The full-text screening was completed by both the primary researcher (PK) and the second reviewer (DW) in December 2021. Data extraction and CASP were initially completed by the primary researcher (PK) and reviewed by the second reviewer (DW). The third reviewer, also the primary supervisor of this project (CM), was assigned to resolve conflicts after discussions at each stage. However, no conflicts were present throughout the stages of progress. Therefore, the third reviewer’s involvement was not required in the process. At the completed full-text screening stage, five studies met the eligibility criteria to be included in the review (Figure 1). Considering the low number of studies (n=5) to be included, the researcher consulted with supervisors to seek advice to proceed. The consensus was reached to expand to include more gray literature. The search results yielded from the gray literature search and selection process of results are shown in Figure 2.

### Data Analysis and Presentation

The findings of this scoping review will be presented in a tabular format. The articles included in the review will be summarized and displayed in categories describing the studies included in the full review. Analysis of the results will consist of a narrative synthesis to link the results and research objectives using thematic analysis guided by Braun and Clarke [31], a commonly used technique suitable for analyzing qualitative literature. The data analysis will be carried out by the primary researcher (PK) and will be validated by the primary (CM) and secondary (DW) supervisors. The template to extract the relevant data from the included studies and the description of information that will be extracted are presented in Textbox 3.

**Template for data extraction.**

**Study**

Description of information being extracted: the title and reference of the study

**Country**

Description of information being extracted: identify the country in which the study was conducted

**Design**

Description of information being extracted: details of the study design, methodology, and research frameworks

**Participants**

Description of information being extracted: details of the participants, number of participants, age, etc.

**Main findings**

Description of information being extracted: results of the study, highlights, limitations, and conclusions

## Results

An initial search of the databases was conducted in October 2021. After consulting with the supervisory team (CM and DW) for this project to continue, the search was completed in December 2021. We anticipate that the full scoping review will be completed in December 2022, as this scoping review is part of a qualification that the researcher PK is currently undertaking. The full scoping review is anticipated to be submitted for publication in December 2022.

The scoping review includes 5 articles in the initial review. The process of finding the articles to be included in the review is presented in Figure 1, which shows the PRISMA-ScR flowchart. Of the final articles included, one article is a study performed in NZ, while the others were performed in Australia. All five studies included in the scoping review were qualitative studies conducted by interviewing participants and had the common aim of understanding perspectives. Four of the articles explored medical cannabis–related perspectives, while one explored the use of opioids in the population of pharmacists. Although this study is not directly related to medical cannabis perspectives for pharmacists, the themes present in the study are associated with the aim of this scoping review and will contribute valuable insights to the discussion.

The databases Australia/New Zealand Reference Centre, Informit New Zealand Collection, INNZ: Index New Zealand, ProQuest Dissertations & Theses Global, and AUT Tuwhera Research Repository were searched along with the website nzresearch.org.nz. This website enabled searching of theses, dissertations, and academic articles produced at educational institutes throughout NZ. The databases did not reveal relevant information specific to this scoping review. The search for gray literature was performed after consultation with supervisors to find more texts relevant to the aim to be included in the full review. However, the search results were unable to yield results related to the topic of this review (n=0). Given the limited timeframe to complete the scoping review as part of a qualification for the primary author (PK), the supervisory team agreed that data saturation was reached at this stage of the literature search.

## Discussion

To our knowledge, this is the first scoping review to explore legal cannabis users and health professional perspectives to treat chronic pain with medical cannabis in NZ and Australia since the rollout of medical cannabis in both countries. Studies that have been previously conducted on medical cannabis occurred before medical cannabis legalization and mostly used surveys [17,32-36]. This makes our review focusing on regulated and prescribed medical cannabis products using interviews unique as it examines perspectives to develop themes that highlight the research gaps and challenges to accessing medical cannabis relevant to NZ. The developed themes discussed in this scoping review may highlight why medical cannabis users prefer medical cannabis over other first-line treatments and aid in improving medical cannabis treatment for chronic pain in NZ.

This scoping review highlights the current barriers for patients using medical cannabis through their health system experience. These outlined barriers may assist in proposing recommendations that may guide policy shifts in NZ and suggest future study directions. These suggestions will be formed from the themes that arise from this scoping review results, such as spillover effects of medical cannabis, the impact of medical cannabis on the youth, the existing barriers to accessing medical cannabis treatment, for example, the associated treatment costs, the limited knowledge of health professionals to recommend medical cannabis treatment, and the stigma associated with medical cannabis use.

The suggestions and recommendations in this review will focus on highlighting and addressing the barriers identified through perspectives to improve the future of accessing medical cannabis in NZ. Suggestions may include improvements to education of health professionals in other countries and the effects of education in medical practice, for example, achieving higher prescription rates to access medical cannabis for users through the health system that may improve access to medical cannabis. Increasing education among health professionals and increasing access to medical cannabis using the health system may also reduce the stigma surrounding medical cannabis use. Other suggestions may include conducting a study examining the perspectives of health professionals and legal CBD users in NZ to understand the reasons for using medical cannabis. A future study in this direction may highlight the challenges experienced by health professionals and medical cannabis users in NZ. Therefore, studying the perspectives of health professionals and medical cannabis users to suggest recommendations may aid in informing knowledge for NZ policy makers that may provide better access to medical cannabis and guide future policy making on medical cannabis in NZ.

The future directions of this study will include suggesting the current research that the primary author (PK) is undertaking, which includes interviewing medical cannabis users and health professionals to examine CBD user perspectives in NZ and highlight their experiences of using medical cannabis through the NZ health system. This research will follow the suggestions of this scoping review and interview participants that may provide valuable information to guide policy reformation of medical cannabis in NZ.

### Limitations

The inclusion criteria for this scoping review are limited to studies that use qualitative methods. Therefore, the results may not represent the broader population of perspectives studied using other methods, for example, surveys. However, this scoping review will recommend a future study in NZ that uses interviews to add knowledge that examines the reasons for using medical cannabis in NZ. We also recognize that limiting the scoping review to include studies from only NZ and Australia may not represent the body of knowledge that exists on medical cannabis user and health professional perspectives globally. However, it is essential to note that this scoping review aims to provide suggestions for policy recommendations in NZ. Therefore, it contributes to the currently limited evidence for the country for which recommendations are being suggested.

### Conclusions

The conclusions of this scoping review will summarize the importance of medical cannabis and the reasons for its use among medical cannabis users and health professionals using perspectives. A summary of the evidence discussed will be an important component to reinforce the success of addressing the overall aim. The challenges highlighted through the discussions of this scoping review include access to medical cannabis due to the associated treatment costs, the limited knowledge of health professionals to recommend medical cannabis treatment for chronic pain, and the stigma associated with its use.

These elements indicate that using perspectives to understand the reasons for medical cannabis use may enforce policy changes that address the current challenges in NZ for chronic pain treatment with medical cannabis. Through this scoping review, the importance of implementing interventions that educate health professionals to increase access to medical cannabis using the health system may address a significant barrier, for example, the limited knowledge of health professionals to prescribe medical cannabis. Additionally, this scoping review may suggest introducing a wider selection of medical cannabis products into the NZ market. Introducing new products may reduce the associated costs of treatment through competition and will increase product variety for consumers that are currently limited.

Highlighting the concerns raised by health professionals and medical cannabis users may reinforce the importance of medical cannabis research using perspectives. The same is demonstrated through the overall aim of this scoping review that highlights the importance of understanding perspectives to contribute to effecting a positive change for the future policy reformation of medical cannabis in the context of NZ. Future research directions undertaken by the primary author (PK) will be summarized. The summary will acknowledge the contribution of the research currently being undertaken as a valuable contribution to advancing medical cannabis research using perspectives in NZ that may contribute to future policy reformation for medical cannabis in NZ.

## Appendix

Multimedia Appendix 1 Search string used to perform the search for published literature in electronic databases.

## Abbreviations

CASP: Critical Appraisal Skills Programme
CBD: cannabidiol
CNS: central nervous system
NZ: New Zealand
PRISMA-ScR: Preferred Reporting Items for Systematic Reviews and Meta-Analyses Extension for Scoping Reviews
THC: delta-9-tetrahydrocannabinol
