# Supplementary material for: Analyzing the Perspectives of Health Professionals and Legal Cannabis Users on the Treatment of Chronic Pain With Cannabidiol: Protocol for a Scoping Review
Source: JMIR Res Protoc. 2023 Jan 20;12:e37697. doi: 10.2196/37697 (PMC9898834; doi:10.2196/37697)
Supplement: Multimedia Appendix 1 [file resprot_v12i1e37697_app1.pdf]

MY EBSCOhost

Monday, August 08, 2022 4:36:36 AM

#	Query	Limiters/Expanders	Last Run Via	Results
S5	(cbd oil or cannabidiol or cannabis or medical marijuana) AND (pain or chronic pain or longterm pain or persistent pain)	Expanders - Apply related words; Apply equivalent subjects<br>Search modes - Boolean/Phrase	Interface - EBSCOhost Research Databases<br>Search Screen - Advanced Search<br>Database - CINAHL Complete;MEDLINE	4,649
S4	(cbd oil or cannabidiol or cannabis or medical marijuana) AND (perspectives or views or perceptions or attitudes or opinion) AND (pain or chronic pain or longterm pain or persistent pain)	Expanders - Apply related words; Apply equivalent subjects<br>Search modes - Boolean/Phrase	Interface - EBSCOhost Research Databases<br>Search Screen - Advanced Search<br>Database - CINAHL Complete;MEDLINE	676
S3	(cbd oil or cannabidiol or cannabis or medical marijuana) AND (perspectives or views or perceptions or attitudes or opinion)	Expanders - Apply related words; Apply equivalent subjects<br>Search modes - Boolean/Phrase	Interface - EBSCOhost Research Databases<br>Search Screen - Advanced Search<br>Database - CINAHL Complete;MEDLINE	6,147
S2	(cbd OR cannabidiol) AND ((chronic pain) OR (non cancer chronic pain))	Limiters - Published Date: 20100101-20211231<br>Expanders - Apply related words; Apply equivalent subjects<br>Search modes - Boolean/Phrase	Interface - EBSCOhost Research Databases<br>Search Screen - Advanced Search<br>Database - CINAHL Complete;MEDLINE	Display
S1	(cbd OR cannabidiol* OR (medicinal cannabis) OR (medical	Expanders - Apply related words; Apply equivalent subjects	Interface - EBSCOhost Research Databases<br>Search Screen - Advanced	Display

	marijuana)) AND (Pain OR (Non cancer chronic pain) OR (Chronic Pain) OR (Neuropathic pain) OR (chronic condition*)) AND (perspective* OR opinion* OR attitude* OR view* OR experience* OR (lived experience*) OR knowledge) AND (professional* OR (health professional*) OR (medical professional*) OR user* OR patient* OR doctor*) AND (NZ OR (New Zealand) OR Aotearoa OR Australia OR Auz OR Aus)	Search modes - Boolean/Phrase	Search Database - CINAHL Complete;MEDLINE
